# The shed P2X7 receptor is an index of adverse clinical outcome in COVID-19 patients

**DOI:** 10.3389/fimmu.2023.1182454

**Published:** 2023-05-05

**Authors:** Valentina Vultaggio-Poma, Juana Maria Sanz, Andrea Amico, Alessandra Violi, Sara Ghisellini, Stefano Pizzicotti, Angelina Passaro, Alberto Papi, Marco Libanore, Francesco Di Virgilio, Anna Lisa Giuliani

**Affiliations:** ^1^ Department of Medical Sciences, University of Ferrara, Ferrara, Italy; ^2^ Department of Chemical, Pharmaceutic and Agricultural Sciences, University of Ferrara, Ferrara, Italy; ^3^ Department of Translational Medicine and for Romagna, University of Ferrara, Ferrara, Italy; ^4^ Laboratory of Clinical Pathology, St. Anna Hospital, Ferrara, Italy; ^5^ Infectious Diseases Unit, St. Anna Hospital, Ferrara, Italy

**Keywords:** COVID-19, shed P2X7R, shed NLRP3, disease progression, circulating markers

## Abstract

**Introduction:**

The pathophysiology of the Corona Virus Disease 2019 (COVID-19) is incompletely known. A robust inflammatory response caused by viral replication is a main cause of the acute lung and multiorgan injury observed in critical patients. Inflammasomes are likely players in COVID-19 pathogenesis. The P2X7 receptor (P2X7R), a plasma membrane ATP-gated ion channel, is a main activator of the NLRP3 inflammasome, of the ensuing release of inflammatory cytokines and of cell death by pyroptosis. The P2X7R has been implicated in COVID-19-dependent hyperinflammation and in the associated multiorgan damage. Shed P2X7R (sP2X7R) and shed NLRP3 (sNLRP3) have been detected in plasma and other body fluids, especially during infection and inflammation.

**Methods:**

Blood samples from 96 patients with confirmed SARS-CoV-2 infection with various degrees of disease severity were tested at the time of diagnosis at hospital admission. Standard haematological parameters and IL-6, IL-10, IL-1β, sP2X7R and sNLRP3 levels were measured, compared to reference values, statistically validated, and correlated to clinical outcome.

**Results:**

Most COVID-19 patients included in this study had lymphopenia, eosinopenia, neutrophilia, increased inflammatory and coagulation indexes, and augmented sNLRP3, IL-6 and IL-10 levels. Blood concentration of sP2X7R was also increased, and significantly positively correlated with lymphopenia, procalcitonin (PCT), IL-10, and alanine transaminase (ALT). Patients with increased sP2X7R levels at diagnosis also showed fever and respiratory symptoms, were more often transferred to Pneumology division, required mechanical ventilation, and had a higher likelihood to die during hospitalization.

**Conclusion:**

Blood sP2X7R was elevated in the early phases of COVID-19 and predicted an adverse clinical outcome. It is suggested that sP2X7R might be a useful marker of disease progression.

## Introduction

Corona Virus Disease 2019 (COVID-19), caused by the Severe Acute Respiratory Syndrome Coronavirus 2 (SARS-CoV-2), is characterized by a massive inflammatory response that is the main cause of the acute lung injury and of the other complications observed in more critical patients (1–3). While fever and respiratory tract distress are the most common symptoms, extra-respiratory clinical manifestations in other organs/systems may also occur occasionally and be the signs of presentation (4). Eventually, critical patients may develop severe complications including multiple organ failure (MOF). A thorough understanding of the pathogenesis, as well as the identification of reliable markers of disease progression, is crucial to establish a timely therapy.

Macrophages, as a main source of inflammatory mediators, play a key role in the response to SARS-CoV-2 infection. Notably, in severe COVID-19 lung macrophages undergo a virus-triggered uncontrolled activation reminiscent of the macrophage activation syndrome (MAS) observed in some rheumatological diseases (5, 6). This unchecked macrophage activation is one of the causes of the cytokine storm syndrome typical of severe COVID-19 patients. Several key pro-inflammatory, but quite interestingly also anti-inflammatory, cytokines are released to a high level during COVID-19-associated cytokine storm (7, 8). Release of pleiotropic cytokines of the IL-1 and IL-18 family is widely thought to be due to direct or indirect activation of the NLRP3 inflammasome by SARS-CoV-2 (9, 10), but these cytokines are not always increased in COVID-19 patients (11). NLRP3-independent cytokines, such as IL-2, IL-6, TNF-α and IFN-γ are more commonly elevated and extensively involved in disease pathogenesis (10). The IL-10 case is intriguing as increase of this cytokine in COVID-19 is widely believed to be the result of a feed-back mechanism. However, this might be a simplistic interpretation overshadowing a so far unanticipated pro-inflammatory activity of IL-10. Scattered reports show that administration of pegylated recombinant human IL-10 triggered immune activation in cancer patients with encouraging results in cancer control (12, 13). Based on this evidence, it has been recently proposed that the sustained increase of IL-10 blood levels might be directly involved in the progression and aggravation of COVID-19 by stimulating release of additional cytokines involved in the “cytokine storm” and/or by accelerating T cell exhaustion (8).

The SARS-CoV-2 spike protein binds and exploits the ACE2 protein, a widely expressed plasma membrane receptor, as a main cell entry pathway (14). In macrophages, the spike protein also binds TLR4 and stimulates the associated pro-inflammatory responses (15). SARS-CoV-2-challenged cells release intracellular DAMPs (damage associated molecular patterns) (16) that, together with viral products, support macrophage hyperactivation and the release of inflammatory mediators. One of the earliest and most ubiquitous DAMPs released at all inflammatory sites is ATP (17). Extracellular ATP (eATP) binds and activates P2 receptors (P2R), among which the P2X7 receptor (P2X7R). This P2R subtype is highly expressed by immune cells, is a potent activator of the NLRP3 inflammasome, promotes IL-1β and IL-18 release, and has a strong cytotoxic and pro-inflammatory activity (18–22). Pyroptosis, a P2X7R- and NLRP3-dependent inflammatory cell death, is suggested to contribute to COVID-19 disease severity (23, 24).

Monocytes and macrophages express high levels of both ACE2 and P2X7R, thus are very likely to be over-activated by the co-stimulation with both the virus and eATP. Thus, the P2X7R already known to be involved in the pathogenesis of lung diseases, might be an appealing target in COVID-19 (25–28).

Recently, a shed form of the P2X7R (sP2X7R) has been detected in peripheral blood, partly associated to circulating microparticles (MPs) (29). Blood levels of sP2X7R correlated with those of the acute phase reactant C-reactive protein (CRP) and were increased in various inflammatory conditions (29, 30), COVID-19 included (31). Shed NLRP3 (sNLRP3) has also been detected in human fluids in different inflammatory pathologies (32–35) and proposed to be a marker of disease severity (36). Of interest, P2X7R activation triggers release of IL-6 *via* different mechanisms including mechanical stress, reactive oxygen species (ROS) production, and ion fluxes (37–42) while its blockade has been associated to inhibition of IL-10 secretion (43). Both cytokines are over expressed and associated to a bad prognosis in COVID-19 patients (8, 44).

In this study blood levels of sP2X7R measured at the time of diagnosis at hospital admission in a cohort of COVID-19 patients were correlated to (a) several markers of inflammation and tissue damage, (b) sNLRP3, IL-6, IL-10, and IL-1β levels, and most importantly, (c) disease progression. The main finding was that sP2X7R correlates with pro-calcitonin (PCT) and IL-10 blood levels and allows prediction of disease progression and poor clinical outcome.

## Methods

### Patients

A total of 96 patients were screened at time of admission at the S. Anna University Hospital (Ferrara, Italy) between April 30 and November 30, 2020. All patients were diagnosed with COVID-19 of various degrees of severity. Patients’ characteristics are reported in **Tables 1**, **2**. Blood samples were collected in vacutainers with or without EDTA, at time of diagnosis soon after hospital admission. Whole blood samples were employed to measure haematological parameters as below. Sera samples were obtained following centrifugation at 4°C for 15 min at 1000xg, aliquoted and stored at -80C° until use. Freezing/thawing cycles were carefully avoided. All subjects gave written informed consent in accordance with the Declaration of Helsinki. The protocol was approved by the Ethical Committee of Ferrara district (Study 386/2020/Oss/UniFe). Demographic and clinical data, comorbidities at admission and COVID-19 related parameters were duly registered.

**Table 1:** Patients’ demographic and clinical data at admission.

	Median (Q1-Q3)
	N (%)
Age (years)	71 (54–82)
Sex Male	59 (61.4%)
Systolic blood pressure at admission (mmHg)	130 (120–140)
Diastolic blood pressure at admission (mmHg)	80 (70–90)

**Table 2:** Patients’ comorbidities at admission.

	Frequency
**Hypertension**	57 out of 96 (58.8%)
**Dementia**	28 out of 95 (28.9%)
**Diabetes**	21 out of 96 (21.6%)
**Diabetes with organ damage**	11 out of 93 (11.2%)
**Diabetes without organ damage**	9 out of 94 (9.3%)
**Heart failure**	15 out of 96 (15.5.%)
**Ischemic heart disease**	14 out of 96 (14.4%)
**Stroke or TIA**	10 out of 96 (10.3%)
**COPD**	10 out of 96 (10.3%)
**PCOA**	4 out of 95 (4.1%)
**Localized or hematological tumor**	17 out of 96 (17.5%)
**Metastatic tumor**	5 out of 95 (5.2%)
**Chronic kidney failure III/IV/V stage**	8 out of 96 (8.2%)
**Moderate liver disease**	3 out of 96 (3.1%)
**Mild liver disease**	2 out of 96 (2.1%)
**Peptic ulcer**	2 out of 96 (2.1%)
**Hemiplegia**	2 out of 96 (2.1%)

TIA, transient ischemic attack. PCOA, chronic peripheral obstructive arteriopathy.

COPD, chronic obstructive pulmonary disease.

### Haematological parameters and analytes

The following haematological parameters were measured at the Clinical Pathology Laboratory of the S. Anna University Hospital with standard methods: leukocyte, neutrophil, lymphocyte, monocyte, eosinophil, basophil, and platelet numbers; pro-thrombin Time (PT) as International Normalized Ratio (INR), activated pro-thrombin time (APTT), fibrinogen, d-dimer, ferritin, high-sensitive C reactive protein (hs-CRP), erythrocyte sedimentation rate (ESR), creatinine, estimated glomerular filtration rate (eGFR), pro-calcitonin (PCT), alanine transaminase (ALT), creatin phosphokinase (CPK), lactate dehydrogenase (LDH), brain natriuretic peptide (BNP) and ultra-sensitive troponin. IL-1β, IL-6, IL-10, sP2X7R and sNLRP3 concentrations were measured following manufacturers’ instructions with the following ELISA kits: Human IL-1β/IL-1F2 Quantikine ELISA kit (SLB50 Biotechne, Minneapolis, MN, USA); Human IL-6 Quantikine ELISA kit (S6050 Biotechne); Human IL-10 Quantikine ELISA kit (S1000B Biotechne); Human P2X purinoceptor 7 (P2RX7) ELISA kit (CSB-EL017325HU Cusabio, Houston, Texas, USA); Human NACHT, LRR and PYD domains-containing protein 3 (NLRP3/Corf7/CIAS1/NALP3/PYPAF1) ELISA kit (CSB-E15885H Cusabio). Optical density was measured at 450 nm with wavelength correction at 570 nm with a Multiskan FC spectrophotometer (Thermo Scientific, Waltham, MA, USA). The reference limits for these analytes were obtained from references reported in the ELISA kits (IL-6, IL-10 and IL-1β), or from previous publications (sP2X7R ref (29) and sNLRP3 ref (32)).

### Statistics

Continuous variables were analysed for normal distribution using Shapiro–Wilk tests and expressed as median and interquartile range (IQR) for not-normally distributed variables. Comparisons between groups were performed with Mann-Whitney U test and among groups with Kruskal-Walli’s test. Categorical variables were presented as counts and percentages and compared with exact Fisher or chi-squared tests.

Spearman rank correlation was used to assess the bivariate association between the parameters of interest. A two-sided p-value ≤0.05 was considered statistically significant. All analyses were performed using SPSS v. 25.0 statistical software (International Business Machines, New York, NY, USA). Missing data for each variable of interest did not exceed 5%.

## Results

### Patients’ characteristics at admission and during hospitalization

Ninety-six COVID-19 patients, 59 males and 37 females with a median age of 71 years, with various degrees of COVID-19 severity were included in this study (**Table 1**). Main comorbidities at admission are shown in **Table 2**. Most patients (58.8%) were affected by hypertension. Dementia and diabetes, the latter with or without organ damage, were also present with high frequency (28.9% and 21.6%, respectively). Other relevant pathologies were heart failure (15.5%), ischemic heart disease (14.4%), transient ischemic attack (TIA) or stroke (10.3%), and haematological/localized (17.5%) or metastatic (5.2%) cancer. Chronic obstructive pulmonary disease (COPD), or chronic peripheral obstructive arteriopathy (CPOA) were present in 10.3 and 4.1% of patients, respectively, whereas chronic kidney failure was present in 8.2% of patients.

At admission, 9.3% of the patients showed gastrointestinal symptoms/signs, 14.4% fever, 25.8% respiratory symptoms/signs, while 43.3% both fever and respiratory symptoms (**Table 3**). During hospitalization, 40.2% of patients were transferred to the Pneumology division, 26.8% required mechanical ventilation, and 13.4% were transferred to intensive care unit (ICU). Finally, 10.3% of patients died during hospitalization, and 24.7% after hospital discharge (**Table 3**).

**Table 3:** COVID-19 related parameters at admission and during hospitalization.

	Frequency
**Fever without other symptoms at admission**	14 out of 94 (14.4%)
**Respiratory symptoms and signs at admission**	25 out of 95 (25.8%)
**Fever and respiratory symptoms and signs at admission**	42 out of 95 (43.3%)
**Gastrointestinal symptoms and signs at admission**	9 out of 95 (9.3%)
**Transfer to Pneumology division**	39 out of 93 (40.2%)
**Requirement for mechanical ventilation**	26 out of 95 (26.8%)
**Transfer to Intensive Care Unit (ICU)**	13 out of 93 (13.4%)
**Death during hospitalization**	10 out of 96 (10.3%)
**Death within 100 days from admission**	24 out of 90 (24.7%)

### Laboratory parameters and blood sP2X7R and sNLRP3 levels

Patients’ blood samples were analysed. Laboratory and clinical parameters are shown in **Table 4**. Leucocytosis was present in 27.8% and leukopenia in 11.1% of the patients; neutrophilia was present in 31.5% and neutropenia in 7.9% of the patients; lymphopenia and eosinopenia, common findings in COVID-19, were found in 65.2% and 53.9% of patients, respectively. Monocytes were increased in 10.1% and decreased in 6.7% of the patients, while basophils were normal in nearly all patients (94.4%). Thrombocytopenia was present in 17.8% and thrombocytosis in 3.3% of patients. PT(INR) was increased above the reference limit in 21% of patients, APTT in 96.6%. Fibrinogen and d-dimer were elevated in most patients, 72.6% and 68.0%, respectively. CRP was above 3 mg/L in 54.7%, and between 0.5 and 3 mg/L in 32.0% of patients. Inflammatory status of most patients was also witnessed by increased ESR (73.3%) and ferritin (35% of male and 33.3% of female patients; male and female patients were evaluated against their respective reference values). In 42.1% of patients PCT was above 0.5 ng/ml, and in 15.8% PCT values were higher than 2 ng/ml, indicating the presence of infection or sepsis. Creatinine was increased in 22.1% and eGFR was reduced in the majority (73.3%) of patients. ALT was increased in 13.0% of patients (male and female patients were evaluated against their respective reference values), while CPK was elevated in 38.7% of patients. LDH and BNP were elevated in 48.1% and 49.2% of patients, respectively, whereas troponin showed a higher increase in female (57.9%) than in male patients (46.2%) (**Table 4**). Among main inflammatory cytokines, IL-6 and IL-10 were increased in most patients, 61.9% and 79.4%, respectively, whereas IL-1β only in a small percentage (23.9%) (**Table 5**). Very interestingly, sP2X7R and sNLRP3 were significantly increased in 61.1% and 74.0% of patients, respectively (**Table 5**). Differences between men and women were not significant as regard age and blood levels of sP2X7R, sNLRP3, IL-1β, IL-10, and CRP. On the contrary, men showed IL-6 blood levels significantly higher than women (p=0.003) (not shown). However, this finding has no relevance to our data because the percentage of men and women was not significantly different in the different conditions evaluated (presence of fever and respiratory symptoms, transfer to the Pneumology division, need for mechanical ventilation, transfer to the ICU and death during hospitalization).

**Table 4:** Haematological, inflammatory, and coagulative parameters.

	Median (Q1-Q3)	Reference values	% under	% within	% over
**Leukocytes (n cells x10^3^/µl)**	7.8 (5.4-11.8)	4.0 – 11.0	11.1	61.1	27.8
**Neutrophils (n cells x10^3^/µl)**	5.4 (3.3-9.2)	2.0 – 7.0	7.9	60.6	31.5
**Lymphocytes (n cells x10^3^/µl)**	1.3 (0.8-1.8)	1.5 – 5.0	65.2	34.8	0
**Monocytes (n cells x10^3^/µl)**	0.55 (0.39-0.82)	0.2 – 1.0	6.7	83.2	10.1
**Eosinophils (n cells x10^3^/µl)**	0.03 (0.00-0.12)	0.04 – 0.4	53.9	38.2	7.9
**Basophils (n cells x10^3^/µl)**	0.02 (0.01-0.04)	0.01 – 0.1	4.5	94.4	1.1
**Platelets (n cells x10^3^/µl)**	224.5 (162.8-318.8)	150 – 450	17.8	78.9	3.3
**Fibrinogen (mg/dl)**	477.5 (393.0-615.0)	150 – 400	0	27.3	72.7
**PT (INR) (secs)**	1.1 (1.0-1.2)	0.8 – 1.2	0	79	21
**APTT (secs)**	1.0 (0.9-1.0)	0.8 – 1.2	3.4	0	96.6
**d-dimer (mg/dl)**	1.1 (0.5-2.5)	<0.5	0	26.4	73.6
**CRP (mg/L)**	3.8 (1.0-10.7)	0 – 3	13.3 (<0.5)	32 (0.5 – 3)	54.7 (>3)
**ESR (mm/h)**	34.0 (19.0-67.5)	0 – 20	0	26.7	73.3
**Ferritin (ng/ml)**	222.0 (69.5-475.3)	24 – 335 M 11 – 306 F	20 8.3	45 58.4	35 33.3
**PCT (ng/ml)**	0.2 (0.1-0.9)	<0.5 >2	0 0	57.9 84.2	42.1 15.8
**Creatinine (mg/dl)**	1.03 (0.85-1.23)	0.5 – 1.2	0	77.9	22.1
**eGFR (ml/min)**	70.3 (52.3-90.0)	90 – 120	73.3	24.1	2.6
**ALT (U/L)**	20.0 (12.8-34.5)	<50 M <35 F	0 0	83.9 88.2	16.1 11.8
**CPK (U/L)**	97.0 (56.0-197.0)	<145	0	61.3	38.7
**LDH (U/L)**	239.0 (206.0-342.0)	<248	0	51.9	48.1
**BNP (pg/ml)**	109.0 (34.0-243.0)	0 – 100	0	58.8	49.2
**Troponin (ng/L)**	17.0 (5.5-45.0)	<20 M <12 F	0 0	53.8 42.1	46.2 57.9

The analytes were measured using standard methods at the Laboratory division of the S. Anna University Hospital of Ferrara. For ferritin, ALT and troponin different sex-related reference values were employed. For CRP and PCT two different cut-off values, indicating clinical condition worsening, were considered.

PT (INR); pro-thrombin time (international normalized ratio); APTT, activated pro-thrombin time; CRP, C reactive protein; ESR, erythrocyte sedimentation rate; PCT, pro-calcitonin; eGFR, estimated glomerular filtration rate; ALT, alanine transaminase; CPK, creatine phosphokinase; LDH, lactate dehydrogenase; BNP, B natrium peptide.

**Table 5:** Blood levels of sP2X7R, sNLRP3, and cytokines.

	Median (Q1-Q3)	Reference values	% under	% within	% over
**IL-6 (pg/ml)**	18.2 (9.5-42.8)	1.5 – 15	2.1	36	61.9
**IL-10 (pg/ml)**	10.9 (8.2-21.3)	<7.8	0	20.6	79.4
**IL-1β (pg/ml)**	1.6 (0.5-3.5)	<3.9	0	76.1	23.9
**sP2X7R (pg/ml)**	97.4 (50.2-174.4)	16.7 – 82.17	3.2	35.7	61.1
**sNLRP3 (pg/ml)**	250 (60.0-760.0)	9.0 - 49.0	10.4	15.6	74.0

The analytes were measured following ELISA kits manufacturers’ instructions. The reference values for IL-6, IL-10 and IL-1β were obtained from the corresponding ELISA kits. The reference values for sP2X7R and sNLRP3 were those contained in the references [29] and [32], respectively. IL-6: interleukin 6; IL-10: interleukin 10; IL-1β: interleukin 1 β; sP2X7R: soluble P2X7 receptor; sNLRP3: soluble nucleotide-binding oligomerization domain (NOD), leucine-rich repeat (LRR)-containing protein (NLRP) 3.

### sP2X7R and sNLRP3 correlations with hematological, inflammatory, and coagulation indexes

Shed P2X7R at diagnosis negatively and significantly correlated with age (r= -0.244; p = 0.017), and lymphocyte (r = -0.215; p = 0.046), and eosinophil (r = -0.254; p = 0.018) blood count, thus being lower in older patients and higher in patients with lymphopenia and eosinopenia, common laboratory features of COVID-19 patients (**Table 6**). Shed P2X7R did not significantly correlate with CRP but, very interestingly, showed a strong positive correlation with PCT (r = 0.355; p = 0.009), an index of bacterial infection more specific than CRP. Neither sNLRP3, IL-6 nor IL-10 showed a stronger correlation with PCT than sP2X7R. Shed P2X7R also significantly correlated with IL-10 (r = 0.336; p = 0.008), a cytokine very likely implicated in COVID-19 pathogenesis (8) and often elevated in COVID-19 patients (44). Shed P2X7R positively correlated with ALT (r = 0.269; p = 0.012) and negatively with BNP (r = -0.361; p = 0.007), indexes of liver and heart injury or stress, respectively, and, at near significance, with sNLRP3 (r = 0.218; p = 0.057) (**Table 6**).

**Table 6:** Spearman correlation analysis between parameters measured in this study.

	sP2X7R		sNLRP3		IL-6		IL-10		IL-1β	
	r	P-value	r	P-value	r	P-value	r	P-value	r	P-value
age	-0.244	0.017	-0.033	0.774	0.178	0.083	0.189	0.141	0.079	0.451
Leukocytes	-0.040	0.711	0.248	0.038	0.337	0.001	0.253	0.055	0.053	0.624
Neutrophils	-0.019	0.865	0.258	0.032	0.367	<0.001	0.332	0.012	0.105	0.336
Lymphocytes	-0.215	0.046	-0.069	0.574	-0.246	0.020	-0.480	<0.001	-0.158	0.147
Eosinophils	-0.254	0.018	0.074	0.543	0.063	0.558	-0.257	0.054	-0.310	0.004
Monocytes	-0.020	0.853	0.344	0.004	0.302	0.004	-0.028	0.836	0.021	0.847
CRP	0.144	0.184	0.237	0.047	0.603	<0.001	0.358	0.008	0.029	0.812
Ferritin	0.204	0.298	0.686	0.002	0.526	0.002	0.350	0.142	-0.085	0.673
PCT	0.355	0.009	0.178	0.259	0.201	0.134	0.130	0.431	-0.232	0.088
d-dimer	-0.209	0.085	0.288	0.033	0.310	0.009	-0.012	0.934	-0.175	0.160
fibrinogen	0.146	0.180	0.127	0.301	0.316	0.003	0.178	0.184	0.251	0.030
creatinine	0.101	0.338	0.053	0.653	0.204	0.048	0.263	0.041	0.247	0.017
eGFR	-0.039	0.754	-0.148	0.272	-0.329	0.004	-0.305	0.042	-0.207	0.081
ALT	0.269	0.012	0.057	0.636	0.085	0.426	0.157	0.239	0.077	0.480
CPK	-0.073	0.554	-0.085	0.527	0.126	0.282	0.291	0.042	0.239	0.044
LDH	0.187	0.104	0.389	0.002	0.320	0.004	0.206	0.132	-0.105	0.359
BNP	-0.361	0.007	0.185	0.240	0.290	0.026	0.327	0.052	0.010	0.943
sP2X7R			0.218	0.057	0.148	0.153	0.336	0.008	0.021	0.845
sNLRP3	0.218	0.057			0.368	0.001	0.290	0.035	0.157	0.178
IL-6	0.148	0.153	0.368	0.001			0.487	<0.001	0.029	0.781
IL-10	0.336	0.008	0.290	0.035	0.487	<0.001			0.213	0.099
IL-1β	0.043	0.682	0.182	0.115	0.029	0.781	0.213	0.099		

r, Rho di Spearman; significant correlations are in bold. CRP, C reactive protein; PCT, pro-calcitonin; eGFR, estimated glomerular filtration time; ALT, alanine transaminase; CPK, creatine phosphokinase; LDH, lactate dehydrogenase; BNP, B natrium peptide; sP2X7R, soluble P2X7 receptor; sNLRP3, soluble nucleotide-binding oligomerization domain (NOD), leucine-rich repeat (LRR)-containing protein (NLRP) 3; IL-6: interleukin 6; IL-10: interleukin 10; IL-1β: interleukin 1 β.

On the other hand, sNLRP3, positively correlated with several laboratory parameters: total leukocyte (r = 0.248; p = 0.038), neutrophil (r= 0.258; p = 0.032), and monocyte (r = 0.344; p = 0.004) counts, CRP (r = 0.237; p = 0.047), ferritin (r = 0.686; p = 0.002), d-dimer (r = 0.288; p = 0.033), LDH (r = 0.389; p = 0.002), IL-6 (r = 0.368; p = 0.001) and IL-10 (r = 0.290; p = 0.035) levels (**Table 6**).

Of the three major inflammatory cytokines tested in this cohort, IL-6 positively correlated with leukocyte (r = 0.337; p = 0.001), neutrophil (r = 0.367; p = <0.001), and monocyte (r = 0.302; p = 0.004) counts, and with CRP (r = 0.603; p ≤ 0.001), ferritin (r = 0.526; p = 0.002), d-dimer (r = 0.310; p = 0.009), fibrinogen (r = 0.316, p = 0.003), creatinine (r = 0.204; p = 0.048), LDH (r = 0.320; p = 0.004), BNP (r = 0.290; p = 0.026), sNLRP3 (r = 0.368, p = 0.001) and IL-10 (r = 0.487; p ≤ 0.001) levels (**Table 6**). IL-6 negatively correlated with lymphocyte number (r = -0.246; p = 0.020) and with eGFR (r = -0.329; p = 0.004). IL-10 showed significant positive correlation with neutrophil count (r = 0.332; p = 0.012), CRP (r = 0.358; p = 0.008), creatinine (r = 0.263; p = 0.041), CPK (r = 0.291; p = 0.042), sP2X7R (r = 0.336, p = 0.008), sNLRP3 (r = 0.290, p = 0.035) and IL-6 (r = 0.487, p < 0.001), and negatively correlated with lymphocyte count (r = -0.480; p ≤ 0.001) and eGFR (r = -0.305; p = 0.042) (**Table 6**). On the contrary, IL-1β showed significant correlations with only a few of the investigated parameters, that is negative correlation with eosinophil count (r = -0.310; p = 0.004) and positive correlations with fibrinogen (r = 0.251; p = 0.030), creatinine (r = 0.247; p = 0.017), and CPK (r = 0.239; p = 0.044) (**Table 6**).

### sP2X7R and sNLRP3 relationships with clinical disease progression

To further investigate the relevance of sP2X7R and sNLRP3 in COVID-19, we used the Mann-Whitney U test for independent samples to verify the relationships between sP2X7R, sNLRP3, the tested cytokines, CRP, and haematological parameters at diagnosis, with different clinical progression indexes and outcomes.

**Figure 1:**
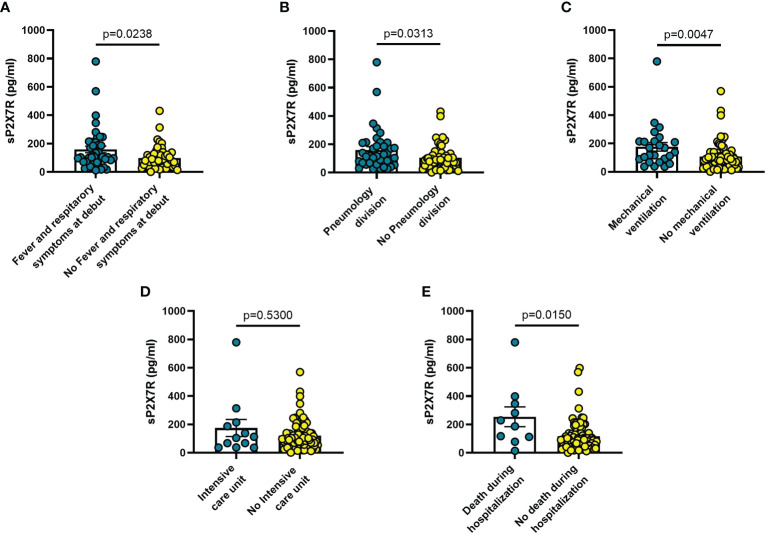
Blood sP2X7R levels vary in different clinical conditions in COVID-19 patients. **(A)** patients with fever and respiratory symptoms at debut (n=42); **(B)** patients who required hospitalization in Pneumology division (n=38); **(C)** patients who required mechanical ventilation (n=25); **(D)** patients who required hospitalization in Intensive care unit (ICU) (n=10); **(E)** patients who died during hospitalization (n=10). Data are means ± SE (standard error). Comparisons between groups were performed using Mann-Whitney U test. A two-sided p-value ≤0.05 was considered statistically significant. sP2X7R: soluble P2X7 receptor.

Blood levels of sP2X7R were significantly higher in patients with fever and respiratory symptoms at diagnosis (n = 42) than in symptom-free patients (n = 51; p = 0.024) (**Figure 1A**), in patients who were transferred to Pneumonology division (n = 38) compared to non-transferred patients (n = 53; p = 0.031) (**Figure 1B**), in patients who required mechanical ventilation (n = 25) versus patients who did not (n = 68; p = 0.005) (**Figure 1C**), and in patients who died during hospitalization (n = 10) versus those who survived (n = 85; p = 0.015) (**Figure 1E**).

**Figure 2:**
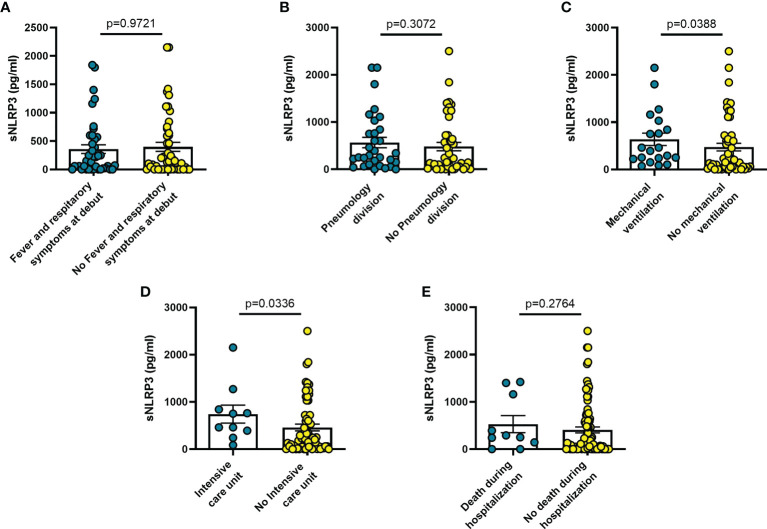
Blood sNLRP3 levels vary in different clinical conditions in COVID-19 patients. **(A)** patients with fever and respiratory symptoms at debut (n=42); **(B)** patients who required hospitalization in Pneumology division (n=38); **(C)** patients who required mechanical ventilation (n=25); **(D)** patients who required hospitalization in Intensive care unit (ICU) (n=10); **(E)** patients who died during hospitalization (n=10). Data are means ± SE (standard error). Comparisons between groups were performed using Mann-Whitney U test. A two-sided p-value ≤0.05 was considered statistically significant. sNLRP3: soluble nucleotide-binding oligomerization domain (NOD), leucine-rich repeat (LRR)-containing protein (NLRP) 3.

On the other hand, sNLRP3 was not significantly different in patients with or without fever or respiratory symptoms at admission (**Figure 2A**), or in patients transferred to Pneumology division (**Figure 2B**). On the contrary, sNLRP3 was significantly higher in patients who required mechanical ventilation (n = 20; p = 0.039) (**Figure 2C**), and in those transferred to the ICU (n = 10; p = 0.034) (**Figure 2D**) even though, despite its higher level in these latter patients, it was not predictive of an early death during hospitalization (**Figure 2E**).

**Figure 3:**
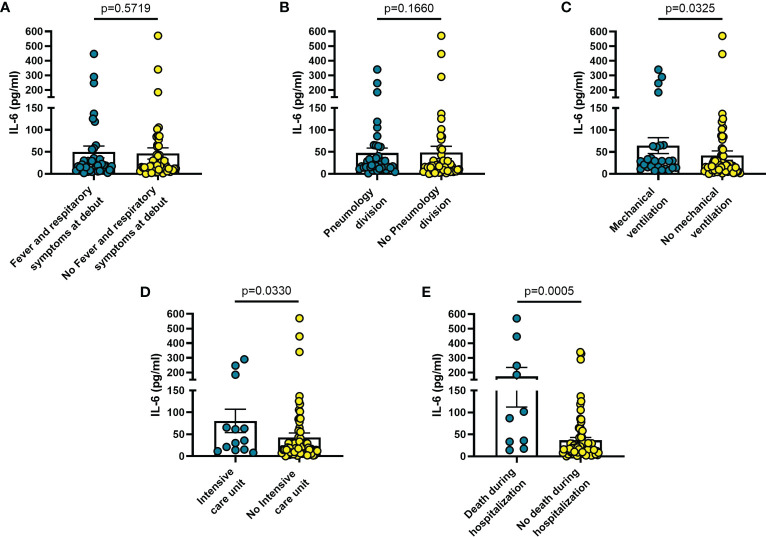
Blood IL-6 levels vary in different clinical conditions in COVID-19 patients. **(A)** patients with fever and respiratory symptoms at debut (n=42); **(B)** patients who required hospitalization in Pneumology division (n=38); **(C)** patients who required mechanical ventilation (n=26); **(D)** patients who required hospitalization in Intensive care unit (ICU) (n=13); **(E)** patients who died during hospitalization (n=10). Data are means ± SE (standard error). Comparisons between groups were performed using Mann-Whitney U test. A two-sided p-value ≤0.05 was considered statistically significant. IL-6: interleukin 6.

**Figure 4:**
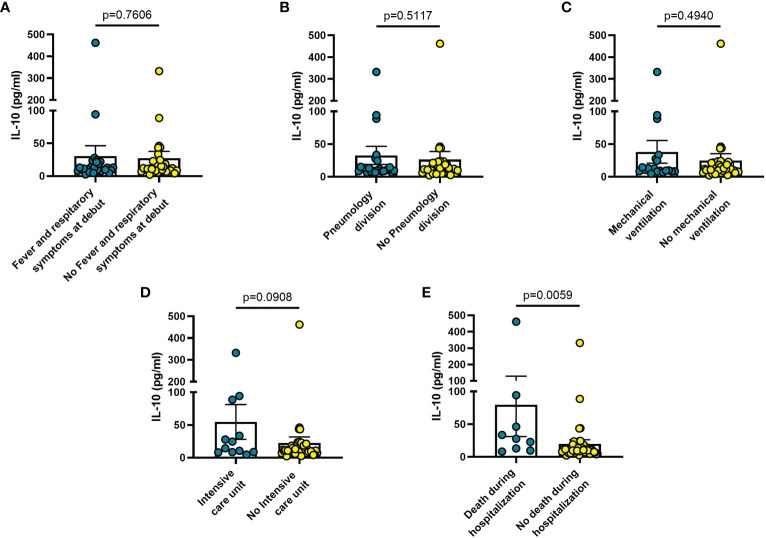
Blood IL-10 levels vary in different clinical conditions in COVID-19 patients. **(A)** patients with fever and respiratory symptoms at debut (n=42); **(B)** patients who required hospitalization in Pneumology division (n=38); **(C)** patients who required mechanical ventilation (n=26); **(D)** patients who required hospitalization in Intensive care unit (ICU) (n=13); **(E)** patients who died during hospitalization (n=9). Data are means ± SE (standard error). Comparisons between groups were performed using Mann-Whitney U test. A two-sided p-value ≤0.05 was considered statistically significant. IL-10: interleukin 10.

**Figure 5:**
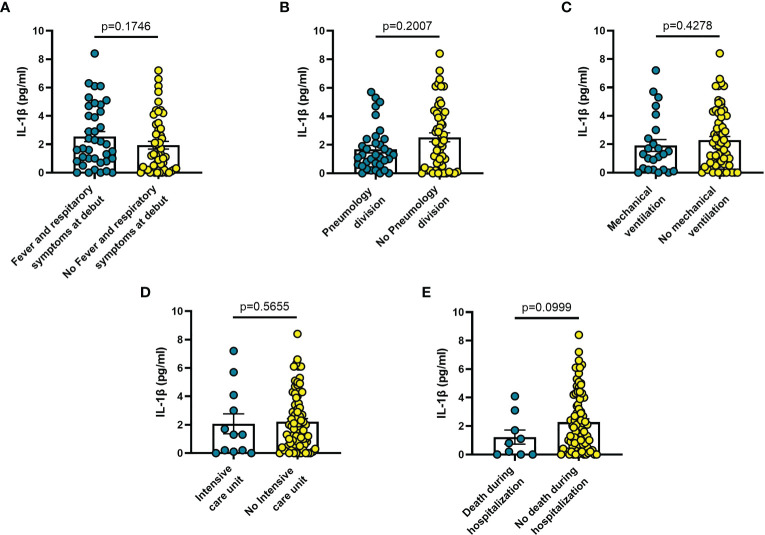
Blood IL-1β levels vary in different clinical conditions in COVID-19 patients: **(A)** patients with fever and respiratory symptoms at debut (n=42); **(B)** patients who required hospitalization in Pneumology division (n=38); **(C)** patients who required mechanical ventilation (n=26); **(D)** patients who required hospitalization in Intensive care unit (ICU) (n=13); **(E)** patients who died during hospitalization (n=9). Data are means ± SE (standard error). Comparisons between groups were performed using Mann-Whitney U test. A two-sided p-value ≤0.05 was considered statistically significant. IL-1β: interleukin 1β.

**Figure 6:**
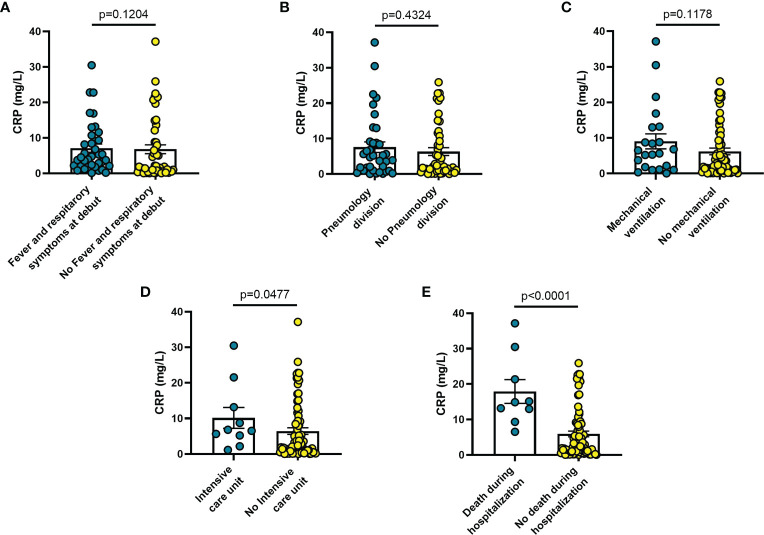
Blood CRP levels vary in different clinical conditions in COVID-19 patients: **(A)** patients with fever and respiratory symptoms at debut (n=42); **(B)** patients who required hospitalization in Pneumology division (n=38); **(C)** patients who required mechanical ventilation (n=26); **(D)** patients who required hospitalization in Intensive care unit (ICU) (n=9); **(E)** patients who died during hospitalization (n=7). Data are means ± SE (standard error). Comparisons between groups were performed using Mann-Whitney U test. A two-sided p-value ≤0.05 was considered statistically significant. CRP: C reactive protein.

Increased blood levels of IL-6 were predictive of the need of mechanical ventilation (n = 26; p = 0.032) (**Figure 3C**), admission to ICU (n = 13; p = 0.033) (**Figure 3D**) or death during hospitalization (n = 10; p = 0.0005) (**Figure 3E**), whereas IL-10 levels were significantly increased only in patients who died during hospitalization (n = 8; p = 0.006) (**Figure 4E**). IL-1β levels showed no significant relationship with any of the clinical conditions considered (**Figure 5**). Finally, CRP was significantly increased in patients who needed admission to ICU (n = 9; p = 0.041) (**Figure 6D**) or who died during hospitalization (n = 9; p < 0.0001) (**Figure 6E**).

**Figure 7:**
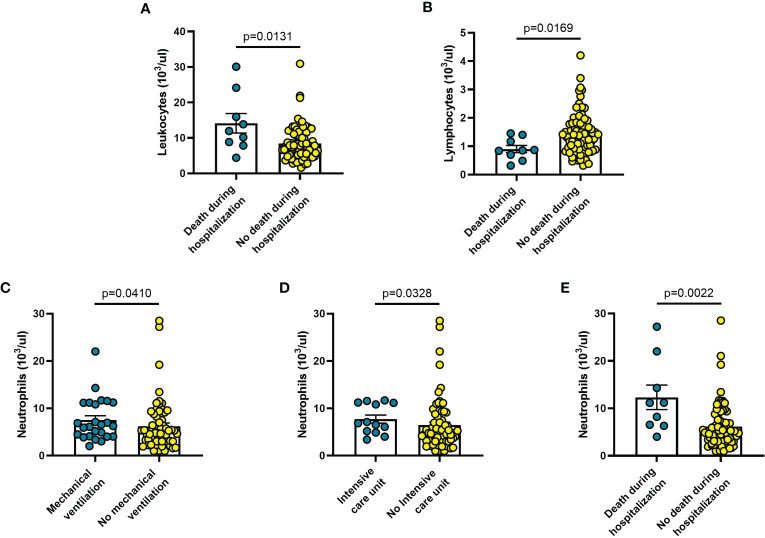
Haematological parameters vary in different clinical conditions in COVID-19 patients: **(A)** patients with fever and respiratory symptoms at debut (n=42); **(B)** patients who required hospitalization in Pneumology division (n=38); **(C)** patients who required mechanical ventilation (n=26); **(D)** patients who required hospitalization in Intensive care unit (ICU) (n=9); **(E)** patients who died during hospitalization (n=9). Data are means ± SE (standard error). Comparisons between groups were performed using Mann-Whitney U test. A two-sided p-value ≤0.05 was considered statistically significant.

Among other haematological parameters measured at diagnosis, the only significant changes were leucocytosis (p = 0.0013) or lymphopenia (p = 0.017) in patients deceased during hospitalization (n = 9) (**Figures 7A, B**), as well neutrophilia in patients who required mechanical ventilation (n = 25; p = 0.041) (**Figure 7C**), admission to ICU (n = 13; p = 0.033) (**Figure 7D**), or deceased during hospitalization (n = 9; p = 0.002) (**Figure 7E**).

The findings shown in **Figures 1**–**6** demonstrate that sP2X7R was the unique analyte significantly elevated in patients with fever and respiratory symptoms at diagnosis (n = 42), and in those who were later hospitalized in the Pneumology division (n = 38). Shed P2X7R, together with sNLRP3, IL-6 and the neutrophil count, was also significantly increased in patients who required mechanical ventilation during their stay in the hospital (n = 26), whereas sNLRP3, IL-6, CRP, and neutrophils were increased in patients transferred to ICU (n = 13). Finally, sP2X7R at diagnosis was increased in patients who died during hospitalization (n = 10). These patients also showed variation of some of the other haematological parameters tested at admission: increased IL-6, IL-10 and CRP, leucocytosis, neutrophilia, and lymphopenia. Therefore, sP2X7R besides being the only analyte significantly elevated at diagnosis in COVID-19 patients with fever and respiratory symptoms, is also an indicator predicting, on par with IL-6, progressive worsening of clinical conditions.

### sP2X7R and sNLRP3 relationships with clinical outcome

We finally investigated sP2X7R, sNLRP3, together with IL-6, IL-10, IL-1β, and CRP blood levels in patients who had three different clinical outcomes: a) survival at 100 days from diagnosis; b) death after hospital discharge within 100 days from diagnosis; c) death during hospitalization.

**Figure 8:**
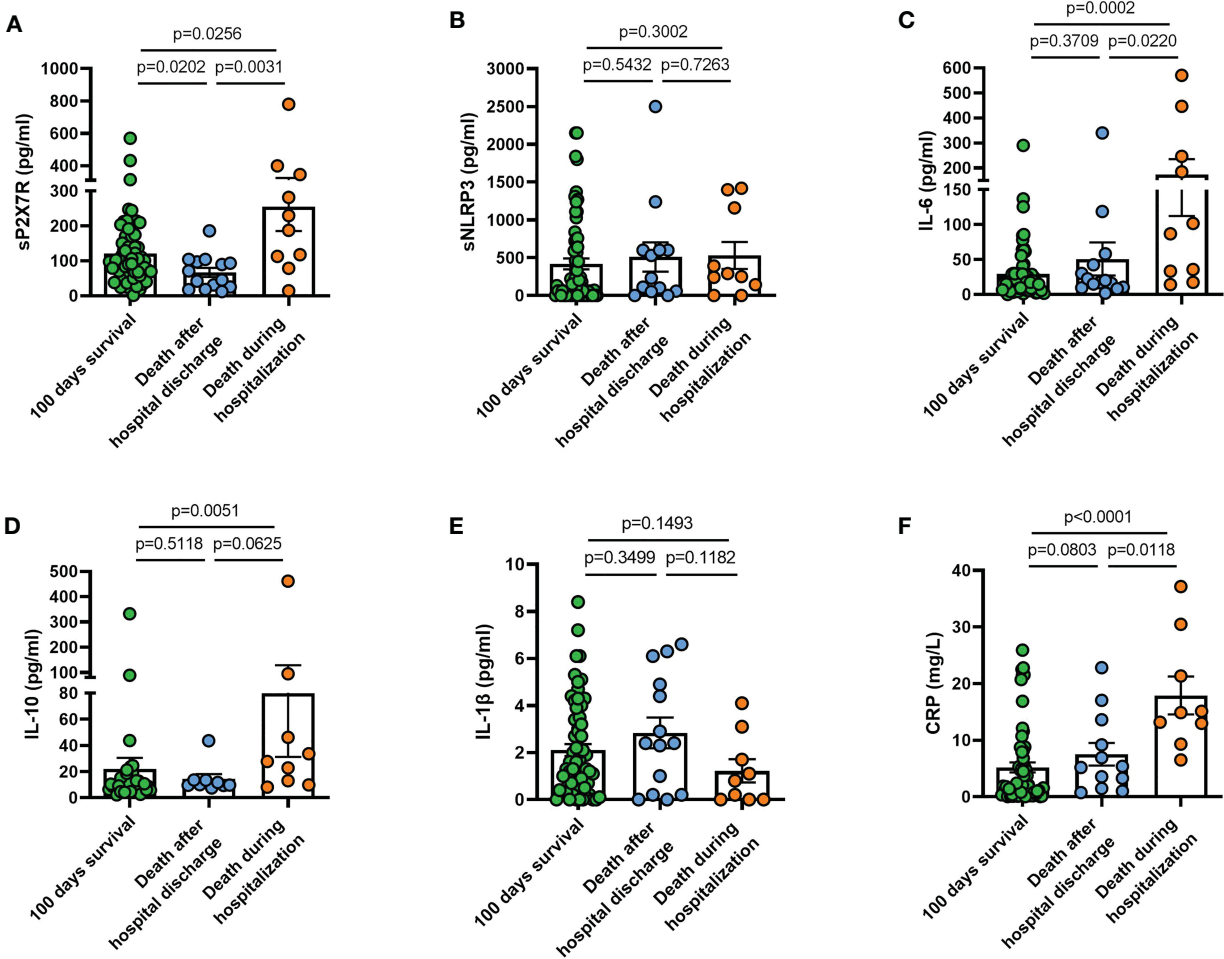
Blood levels at diagnosis of sP2X7R, sNLRP3, IL-6, IL-10, IL-1β, and CRP in patients who had different clinical outcomes. (**A**) sP2X7R was significantly higher in patients who died during hospitalization (n = 10) than in patients who died after hospital discharge within 100 days from diagnosis (n = 14) (p = 0.031), and in patients who survived at least 100 days from diagnosis (n=64) (p = 0.025). sP2X7R was also significantly higher in patients who survived at least 100 days from diagnosis respect to patients who died during hospitalization (p = 0.020). (**B**) sNLRP3 levels were not significantly different between the different groups. (**C**) IL-6 was significantly higher in patients who died during hospitalization respect to patients who survived at least 100 days (p = 0.0002), and respect to patients who died after hospital discharge (p = 0.022). (**D**) IL-10 was significantly higher in patients who died during hospitalization respect to patients who survived at least 100 days (p = 0.005), and at limit of significance (p = 0.062) respect to patients deceased after hospital discharge. (**E**) IL-1β levels were not significantly different between the different groups. (**F**) CRP was significantly higher in patients deceased during hospitalization respect to patients who survived at least 100 days (p < 0.0001), and respect to patients who died after hospital discharge (p = 0.011). Data are means ± SE (standard error). Comparisons between groups were performed using Mann-Whitney U test. A two-sided p-value 0.05 was considered statistically significant. sP2X7R, soluble P2X7 receptor; sNLRP3, soluble nucleotide-binding oligomerization domain (NOD), leucine-rich repeat (LRR)-containing protein (NLRP) 3; IL-6, interleukin 6; IL-10, interleukin 10; IL-1β, interleukin 1 β; CRP, C reactive protein.

Patients who died during hospitalization (n = 10) had significantly higher levels at diagnosis of sP2X7R (p = 0.025), IL-6 (p = 0.0002), IL-10 (p = 0.0051), and CRP (p < 0.0001) than patients who survived at least 100 days from diagnosis (n = 64) (**Figures 8A, C, D, F**). Patients deceased during hospitalization also had significantly more elevated levels of sP2X7R (p = 0.003), IL-6 (p = 0.022), and CRP (p = 0.001), and of IL-10 at limit of significance (p = 0.062), than patients who died after hospital discharge within 100 days from diagnosis (n = 14) (**Figures 8A, C, D, F**). No significant difference was found in sNLRP3, IL-6, IL-10, and CRP levels, except sP2X7R (p = 0.020) between patients who died after hospital discharge and patients who survived at least 100 days from diagnosis (**Figure 8A**). Finally, neither sNLRP3 (**Figure 8B**), nor IL-1 (**Figure 8E**) were significantly different between the groups considered.

## Discussion

The SARS-CoV-2 infection is accompanied by fearful complications due not simply to direct virus-mediated tissue injury, but also to dysregulated hyperinflammation triggered by activation of innate immunity (2). A feature of this condition is the uncontrolled release of inflammatory cytokines such as IL-6, TNF-α, IL-10, IL-1β, and the extensive stimulation of the coagulation pathway (45). Recently, soluble forms of P2X7R and NLRP3 (sP2X7R and sNLRP3) were shown to be elevated in inflammation and sepsis, and tentatively added to the panoply of inflammatory mediators (29, 31, 32). These two molecules are intimately associated as the P2X7R is a potent activator of NLRP3, and both cooperate in promoting maturation and release of IL-1β and IL-18 (18, 46). The P2X7R is an integral membrane receptor, while NLRP3 is a cytoplasmic protein, but their presence in the blood should not be a surprise as both can be shed *via* a MP-mediated mechanism (29, 31, 32). Being both P2X7R and NLRP3 tightly associated to the release of inflammatory cytokines, a reasonable anticipation is that their blood levels in COVID-19 should parallel that of the cytokines typically overexpressed in this disease, e.g., IL-6 and IL-10. In the patient cohort investigated in this study, we found a significant correlation of IL-6 with sNLRP3, but not with sP2X7R. On the other hand, sP2X7R correlated significantly with IL-10, whose activity might rather unexpectedly be related to the potentiation of the inflammatory response aggravating disease course in COVID-19 patients (8).

The main effect of the stimulation of the P2X7R-NLRP3 axis in mononuclear phagocytes is the maturation and release of IL-1β (47), thus we anticipated that increased sP2X7R and sNLRP3 blood levels might correlate with circulating IL-1β. However, IL-1β levels were increased only in a small percentage of patients, with no significant correlation with either sP2X7R or sNLRP3 levels. After all, the role of IL-1β in COVID-19 is not defined and not always correlated with disease severity (11). In our study there was a weak not statisitcally significant correlation between sNLRP3 and sP2X7R blood levels. This suggested to us that blood levels at diagnosis of these two analytes might identify patient subgroups with different clinical outcomes. We therefore split the patient population into six subgroups according to symptom presentation at diagnosis, and to their clinical progression and outcome: 1) patients with fever and respiratory symptoms at hospitalization; 2) patients who needed transfer to the Pneumology division; 3) patients who needed mechanical ventilation; 4) patients who were admitted to ICU; 5) patients who died during hospitalization; 6) patients who deceased within 100 days after hospital discharge. Soluble P2X7R was significantly elevated in patients of all these subgroups, except in patients admitted to ICU, where sP2X7R increase did not reach statistical significance, and in patients who died after hospital discharge. Soluble P2X7R blood levels in this latter group were lower than in the patients who survived longer than 100 days after hospital release, and in the patients who died during hospitalization. Soluble NLRP3 was elevated in patients who needed mechanical ventilation and were admitted to ICU, but not in patients with respiratory symptoms at admission or who died during hospitalization. IL-6 and CRP were elevated at admission in patients who were admitted to ICU and in those who died during hospitalization. Finally, IL-10 was increased only in those patients who died during hospitalization.

In the patient cohort investigated in the present study, sNLRP3, but not sP2X7R, correlated with CRP levels. This is partially at odd with our previous findings showing a correlation between sP2X7R and CRP in a cohort of patients affected by different pathologies (infections, cancer, ischemia and others) (29). In this previous study, the correlation between sP2X7R and CRP was indeed weak in infectious patients (29). On the other hand, in the present study, sP2X7R showed a very strong correlation with PCT. Although the prognostic significance of PCT in COVID-19 is debated (48, 49), it is well known that PCT is a more accurate marker of bacterial infection than CRP, and it was recently found higher in COVID-19 patients with worse clinical progression despite no difference in CRP (50). Therefore, PCT might identify in association with sP2X7R those COVID-19 patients at high risk of developing bacterial complications.

While increased sP2X7R blood concentrations were anticipated given the well-known role of this receptor in inflammation (18) (sP2X7R is elevated in the same percentage of patients as IL-6, i.e. slightly over 60%), the novel finding is that sP2X7R increases were selectively associated with more severe symptoms at admission (fever and dyspnoea), and predicted a worse outcome. These findings expand previous observations by Pelegrin and co-workers reporting a correlation between sP2XR7 plasma levels and COVID-19 severity (31). In addition, we now show that not only sP2X7R correlates with COVID-19 severity but, unique among the analytes investigated, might be an index of the clinical outcome.

The source and mechanism of release of sP2X7R are open questions. We showed previously that the P2X7R can be shed in association with MPs upon stimulation of monocyte/macrophage cells with eATP (29). Circulating mononuclear phagocytes might therefore be a likely source although other blood cell types (e.g., neutrophils, lymphocytes, platelets, erythrocytes) are known to express the P2X7R, and might participate in its release (51). The other open question is the mechanism of shedding. Activation of the P2X7R requires high eATP concentrations, in the high micromolar or low millimolar range. Recent measurements of plasma eATP concentration in COVID-19 patients suggest that this nucleotide might be moderately elevated compared with healthy subjects, and that peripheral blood mononuclear cells release higher amounts upon stimulation (52, 53). Contrariwise, other investigators found lower eATP levels in COVID-19 patients, irrespective of disease severity (54). Even assuming that bulk plasma measurements provide an inaccurate estimate of the eATP concentration at the receptor level, as it is known that its concentration is higher in the pericellular space (55), estimated eATP levels in plasma are well below the threshold for P2X7R activation (56, 57). Several conditions are known to decrease the eATP threshold for P2X7R activation: low Ca^2+^ or Mg^2+^ concentration, alkaline pH, high extracellular K^+^, or co-stimulation with positive allosteric compounds (58–60). In the past, we and others have identified several such compounds active at the P2X7R (61–63). One of the most potent is the antimicrobial cathelicidin LL-37 (62) that is released by inflammatory cells following bacterial and virus infections and recently found increased in the plasma of COVID-19 patients (64). Furthermore, in COVID-19 patients hypomagnesemia is present and correlate to disease severity (65). These observations suggest that in COVID-19 patients several factors might contribute to lower the eATP threshold for P2X7R activation.

The dissociation between sP2X7R and sNLRP3 blood levels is very interesting because it suggests that although these molecules are closely functionally associated and even physically interacting (66), their mechanism of release might be different and dependent on separate stimuli. Lack of correlation with circulating IL-1β suggests that sP2X7R and sNLRP3 shedding is only partially associated to caspase-1 activation and IL-1β processing and release. This is also shown by the low increases of blood IL-1β levels in most patients in this cohort and by the low IL-1β increments in COVID-19 found by other authors (67).

In conclusion, our findings in COVID-19 patients show that increased blood levels of the sP2X7R very interestingly positively correlated with IL-10 and PCT, among the investigated inflammatory markers. Furthermore, increased sP2X7R levels at diagnosis were predictive of critical disease progression and unfavourable outcome.

## Data availability statement

The raw data supporting the conclusions of this article will be made available by the authors, without undue reservation.

## Ethics statement

The studies involving human participants were reviewed and approved by Ethical Committee of Ferrara District, University of Ferrara (Study 386/2020/Oss/UniFe). The patients/participants provided their written informed consent to participate in this study.

## Author contributions 

VVP and AA executed the experiments. SG, AV, SP, AlP, and ML collected the samples. JMS, VVP, and ALG analyzed the data. ALG, and FDV conceived the experiments. ALG, and FDV wrote the paper. VVP, JMS, and ALG prepared the figures. FDV, JMS and ALG provided the funding of the study. ALG, AnP, and FDV provided overall supervision of the study. All authors contributed to the article and approved the submitted version.
